# Probable Autochthonous Transmission of *Mycobacterium lepromatosis* in the Pacific Northwestern United States

**DOI:** 10.4269/ajtmh.26-0034

**Published:** 2026-04-21

**Authors:** Gregory S. Olson, Khalil J. Deveaux, Jason D. Simmons, Joshua A. Lieberman

**Affiliations:** ^1^ Department of Laboratory Medicine and Pathology, University of Washington, Seattle, Washington, USA;; ^2^ Department of Medicine, University of Washington, Seattle, Washington, USA

## Abstract

*Mycobacterium lepromatosis* (*M. lepromatosis*), first described in 2008 as another mycobacterial species that causes Hansen’s disease, has mainly been reported in Mexico, Central America, and the southern United States; however, its disease spectrum and distribution remain unclear. A diagnostically challenging case that represents a likely autochthonous *M. lepromatosis* acquisition in the United States is reported in the present study. A 75-year-old immunocompetent man who had not traveled outside the Pacific Northwest developed an erythematous, ulcerating left thigh plaque, followed by three proximal sporotrichoid nodules. Biopsies revealed granulomatous inflammation with negative acid-fast stain results. Broad-range nontuberculous mycobacterial polymerase chain reaction testing identified *M. lepromatosis* in the sentinel ulcer and possible *Mycobacteroides chelonae* coinfection in the proximal nodule. The patient improved with multidrug therapy. Clinicians in the United States should keep Hansen’s disease in the differential diagnosis, even for patients without international travel. Additional research is needed to clarify the phenotypic spectrum and geographic distribution of this pathogen.

## INTRODUCTION

Hansen’s disease (HD), also known as leprosy, is caused by acid-fast bacilli (AFB) of the nontuberculous mycobacteria (NTM) species *Mycobacterium leprae* (*M. leprae*) and *Mycobacterium lepromatosis* (*M. lepromatosis*).^1^ Humans are the primary carriers, but zoonotic transmission from armadillos has been described for *M. leprae*,^2^^,^^3^ and *M. lepromatosis* has been described in red squirrels in the United Kingdom.^4^ Most HD cases are concentrated in a few countries (e.g., India and Brazil), but evidence is growing for autochthonous *M. leprae* acquisition associated with armadillos in the southeastern United States.^2^^,^^3^^,^^5^^–^^7^ Since the first description of *M. lepromatosis* infection in 2008 by Han et al.,^8^ the majority of infections with this species have been described in Mexico, the southern United States, and Central America;^9^^–^^11^ however, there are increasing reports of local acquisition in the northern United States and Canada.^12^^,^^13^ In the present case report, a challenging diagnostic case of *M. lepromatosis* infection with an unusual presentation confirmed by a broad-range molecular assay is reported. Additional investigation into the acquisition routes and disease spectrum of this mycobacterial species is recommended.

## CASE REPORT

**Figure 1.**
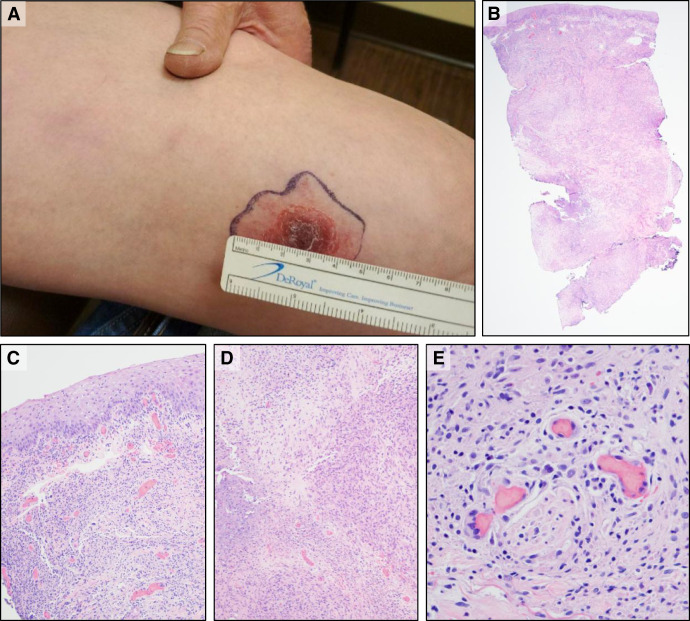
Distal thigh lesion with sporotrichoid nodules. (**A**) April 2021: initial distal thigh lesion and subtle sporotrichoid nodules. (**B**) Punch biopsy from the distal lesion in April revealing a dense mixed inflammatory infiltrate in the superficial and deep dermis (hematoxylin and eosin [H&E], 20×). (**C**) Superficial dermis with multinucleated giant cells and large, ill-formed granulomas (H&E, 100×). (**D**) Deep dermis with areas of necrosis, neutrophilic microabscesses, and large, ill-formed granulomas (H&E, 100×). (**E**) A peripheral nerve encased by granulomatous inflammation without definite neurotropism (H&E, 400×).

In January 2021, a 75-year-old man developed a slightly tender, erythematous plaque on his left distal anterior thigh. A month later, in February, a left inguinal node became enlarged and tender. In March, three more purple and red dermal nodules appeared in a linear, sporotrichoid pattern proximal to the initial lesion, which had progressed into a shallow ulcer by April (Figure 1A). The patient had preexisting bilateral foot paresthesia, which was thought to be unrelated to the new lesions. The neuropathy started acutely ∼10 years previously, immediately after cardiac valve surgery. The patient’s concurrent diabetes mellitus type 2 was mild and diet-controlled (his hemoglobin A1C level was <7% in 2021) and not thought to be the main cause. He declined both electromyography and nerve biopsy. A specific etiology was not established, but symptoms were partially improved with vitamin B12 therapy. He did not complain of any anesthesia associated with his nodular lesions. Sensory mapping with a 1-gram monofilament revealed symmetric sensation in both feet.

In late April, punch biopsies of the shallow ulcer on the left distal anterior thigh and one of the dermal nodules revealed a dense, mixed inflammatory infiltrate in the superficial and deep dermis, including histiocytes and multinucleated giant cells arranged focally in ill-defined granulomas encasing nerves but without definite neurotropism. There were zones of necrosis and neutrophilic microabscesses, but eosinophils were not prominent (Figure 1B–E). Special stains for microorganisms, including periodic acid-Schiff with diastase for fungi, Brown and Brenn for bacteria, and an AFB (Ziehl-Neelsen), yielded negative results.

Biopsies of the distal and proximal thigh lesions were examined with a broad-range assay for NTM at the University of Washington Molecular Microbiology clinical laboratory (Olson et al, 2026; AJTMH-25-0379). Sanger sequencing of a nested *hsp65* polymerase chain reaction (PCR) product from the distal lesion yielded a 125 nt sequence after primer trimming. BLAST analysis revealed 100% identity to published sequences of *M. lepromatosis*, with 100% coverage (EU203593.2, CP083495.1, and CP155806.1 coordinates: 1281678–1281802); the next closest BLAST match was *Mycobacterium decipiens* (OY970459.1) at ∼90% nucleotide identity. Non-nested PCR reactions for 16S rRNA and RNA polymerase B subunit (*rpoB*) produced negative results for mycobacterial DNA. The unexpected results prompted additional testing. DNA was reextracted from the formalin-fixed paraffin-embedded block for repeat nested *hsp65* reactions, which again yielded positive results for *M. lepromatosis.* In total, five of six amplifications yielded positive results across two independent extractions. Given the high confidence taxonomic identification (Supplemental Figure 1) of the trimmed sequence and concerns for analyte degradation during freeze–thaw cycles that would be required, additional species-specific testing was not pursued.

Testing the second proximal lesion with a non-nested PCR for *rpoB* revealed mycobacterial DNA. Sanger sequencing produced a 263 nt product with 100% identity to and coverage of published strains of *Mycobacteroides chelonae* (*M. chelonae*; AY262740); the next nearest BLAST match was *Mycobacteroides saopaulense* (CP010271) at 98.08% identity. Non-nested 16S rRNA PCR and nested *hsp65* PCR results were negative for mycobacterial DNA. Nonsynthetic *M. chelonae* genomic DNA served as a positive control in these assays; therefore, the amplification for the *rpoB* sequence from this organism could reflect either analytical contamination or a true coinfection. In either case, *M. chelonae* templates might have obscured the amplification of *M. lepromatosis* from the proximal lesion.

Given the unexpected identification of *M. lepromatosis*, additional clinical history was gathered; the patient was a dual US–Canadian citizen residing in Washington state who identified as White and non-Hispanic and who had never traveled outside of the Pacific Northwest of the United States. He had a long history of hunting and trapping mink, beaver, and squirrels and helped the Cattlemen’s Association with managing wolves. He had never handled armadillos. He first noticed the distal thigh lesion after wearing waders and denied trapping any animals for at least 3–4 months before its appearance. None of his close contacts had been diagnosed with HD, nor did they have similar lesions. The patient had an extensive cardiac history but no immunosuppression or known immune dysfunction.

**Figure 2.**
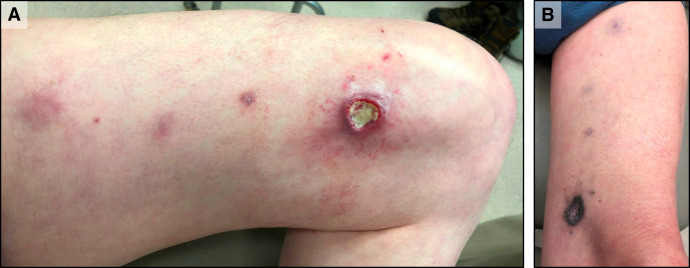
Evolution of distal thigh ulceration with sporotrichoid nodules. (**A**) July 2021: distal thigh with a 2 cm, shallow, moist ulcer with a dark, erythematous, indurated border, central yellow exudate, and three violaceous, 1–2 cm sporotrichoid nodules. (**B**) August 2022: healed distal ulceration with circumferential dense hyperpigmentation and healed nodules with induration and hyperpigmentation.

The biopsy site failed to heal and appeared infected in May, for which the patient received a course of cephalexin and trimethoprim–sulfamethoxazole. The distal ulcer and nodules persisted throughout July (Figure 2A), and a third biopsy of one of the leg lesions proximal to the two previous biopsies revealed continued granulomatous inflammation; however, AFB culture and NTM PCR test results were negative. Standard skin smears collected from six sites, plus a nasal swab, yielded negative results at that time. Antimicrobial selection was complicated by comorbid conditions, including the requirement for anticoagulation (warfarin) and prolonged corrected QT interval due to a dual-chamber automated implanted cardioverter defibrillator with atrioventricular pacing. Because *M. chelonae* could not be excluded as a pathogen and is intrinsically resistant to rifamycins, rifampin was excluded from the regimen. In the absence of mycobacterial growth and drug susceptibility testing, a regimen of clarithromycin at a dose of 500 mg daily, clofazimine at a dose of 50 mg daily, and moxifloxacin at a dose of 400 mg daily was initially chosen, given the predicted activities for both organisms. This regimen required close warfarin monitoring and cardiology follow-up. After ∼1 month, when a history of aortic root dilatation was disclosed, the moxifloxacin was transitioned to minocycline at a dose of 100 mg once daily. This regimen was continued for another 11 months (∼1 year total) with continued clinical improvement and minimal side effects. The cutaneous lesions evolved into hyperpigmented patches (Figure 2B), and the inguinal lymph node shrank to ∼5 mm but remained palpable as a nontender, firm, and mobile nodule.

## DISCUSSION

Recent evidence from ancient DNA suggests that *M. lepromatosis* infected humans across the Americas before European colonization, including a case that occurred in Canada 1,310 years ago.^14^^,^^15^ *Mycobacterium lepromatosis* has been associated with severe forms of diffuse lepromatous leprosy within Mexico,^8^^,^^16^ but little is known about its reservoir and transmission dynamics. The current patient resided in Washington state and had not traveled outside the Pacific Northwest. He presented with sporotrichoid nodules and ulcers with granulomatous inflammation. A broad-range NTM molecular assay was consistent with *M. lepromatosis* infection, and possibly with *M. chelonae* coinfection. Together with recent case reports of a US citizen in the northern Midwest and a Canadian citizen acquiring *M. lepromatosis* without classical risk factors,^13^ the current patient’s acquisition of *M. lepromatosis* in the Northwestern United States emphasizes the need for surveillance of *M. lepromatosis* in northern North America^14^^,^^15^ to better understand transmission routes.

The patient’s history as a hunter and trapper raises the possibility of zoonotic acquisition. Although *M. lepromatosis* is known to infect red squirrels,^4^ the prevalence of *M. lepromatosis* in rodents outside of the British Isles is largely unknown.^17^^,^^18^ The prevalence in animal reservoirs in the Americas requires more comprehensive sampling to better understand susceptible species and the epidemiology. Interestingly, this patient originally attributed his skin lesions to exposure while wearing waders. *Mycobacterium leprae* and *M. chelonae* are known to survive within free-living amoebae,^19^^,^^20^ suggesting another possible route of acquisition. However, the relationship between *M. lepromatosis* and amoebae remains unexplored.

Sporotrichoid nodules have rarely been described during *M. leprae* infection and are usually attributed to the formation of abscesses along a linear nerve tract. In contrast, lymphangitic spread of NTM infections, including *M. chelonae*, is well described.^21^ Notably, only *M. lepromatosis* was detected in the sentinel plaque and ulcer in the distal thigh, whereas only *M. chelonae* was identified in a proximal sporotrichoid nodule. Whether this unusual clinical presentation represents a unique host response to this *M. lepromatosis* strain or is evidence of a concurrent infection or superinfection is unclear. Unfortunately, whole-genome sequencing of the causative organism is unavailable in this case. Future studies are needed to explore genotype–phenotype correlations via comparison with recently published phylogenies,^14^^,^^15^ particularly because there continues to be significant controversy over whether *M. lepromatosis* causes more severe disease (e.g., diffuse lepromatous leprosy or Lucio phenomenon) than *M. leprae.*^1^^,^^8^^,^^9^ Broad-range molecular assays, such as those used in this case (Olson et al., 2026; AJTMH-25-0379), provide powerful tools to limit testing bias to ensure that the full range of phenotypic diversity of a pathogen is identified.^22^^–^^24^

Diagnosis was complicated in the present case by an atypical presentation of paucibacillary disease, negative AFB stain results (although the Ziehl-Neelsen stain used is less sensitive than a Fite stain for *M*. *leprae*, and presumably *M. lepromatosis*), and complex molecular results, emphasizing the need for careful interpretation of PCR-based assays in clinical settings. The reproducibility of the *M. lepromatosis* molecular results, the high quality score of the amplicons, and the lack of obvious sources of *M. lepromatosis* contamination (e.g., control material or other positive samples processed around the same time) all increase confidence in a true analytical positive for *M. lepromatosis* in this case.

Interpreting the molecular detection of *M. chelonae* from one of the sporotrichoid nodules is challenging. The AFB culture results from a proximal lesion (sampled after the initial biopsies but before treatment) remained negative, which could support *M. chelonae* DNA detection representing an analytical contamination. However, inadequate sampling of a paucibacillary infection remains possible, considering that detection occurred at only one of three loci, suggesting a low bacterial burden. Given the atypical clinical presentation and out of an abundance of caution, treatment was initiated to address both pathogens, with good clinical response and limited side effects. The optimal treatment of *M. lepromatosis* remains debated; however, the modified regimen used in this case may offer a strategy for similarly ambiguous cases.

In summary, a case of probable autochthonous acquisition of *M. lepromatosis* in the Northwestern United States is presented. This highlights the need for clinicians in the United States to maintain HD in their differential diagnoses, even for those who have not traveled internationally. Further studies are needed to clarify the geographic distribution, transmission routes (including zoonotic reservoirs), and disease spectrum of *M. lepromatosis*.

## Supplemental Materials

10.4269/ajtmh.26-0034 Supplemental Materials
