# Supplementary material for: Probable Autochthonous Transmission of Mycobacterium lepromatosis in the Pacific Northwestern United States
Source: Am J Trop Med Hyg. 2026 Apr 21;114(6):1078–81. doi: 10.4269/ajtmh.26-0034 (PMC13235588; doi:10.4269/ajtmh.26-0034)
Supplement: Supplemental Materials [file tpmd260034.SD1.pdf]

# Supplemental materials for:

## Probable autochthonous transmission of Mycobacterium lepromatosis in the Pacific Northwest United States

Gregory S. Olson, Khalil Deveaux, Jason D. Simmons, Joshua A. Lieberman

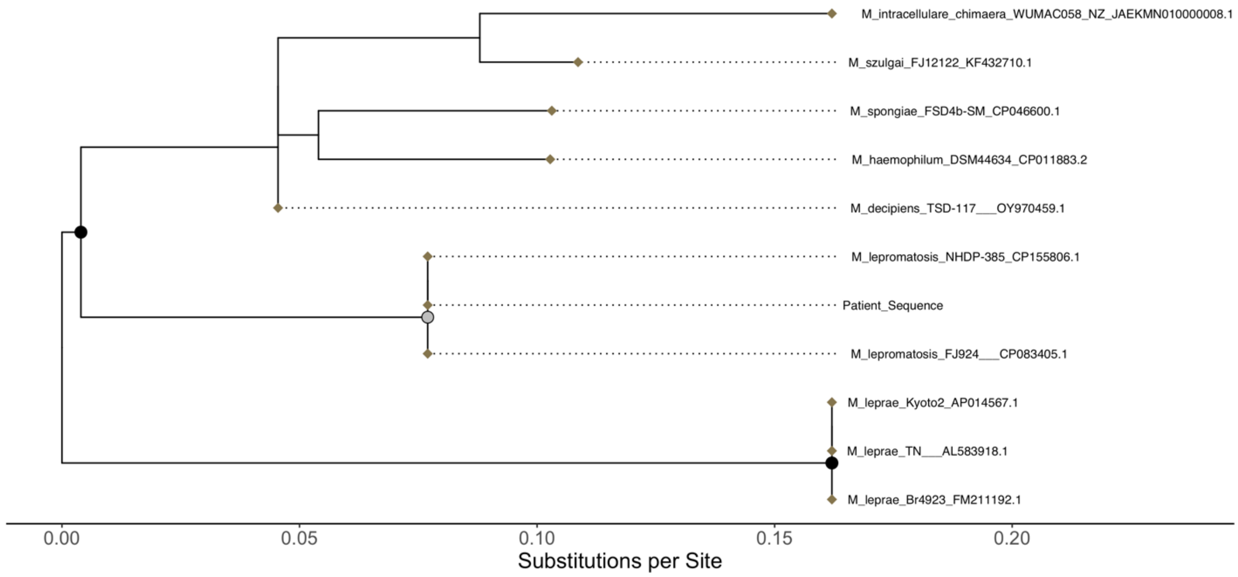


**Supplemental Figure 1. Dendrogram of *Mycobacterium lepromatosis* sequence detected in patient biopsy**. The trimmed, 125 nt partial *hsp65* sequence detected in the patient biopsy was MUSCLE-aligned to representative sequences from *M. lepromatosis*, *M. leprae*, and the most closely related sequences identified by BLAST analysis representing *M. decipiens*, *M. haemophilum*, *M. spongiae*, *M. szulgai*, and *M. intracellulare*. Bootstrap support for the *M. lepromatosis* clade was 91% (Grey-filled node). Black nodes indicate ≥95% bootstrap support. Tree file was generated in IQ-Tree[1] and the figure generated with ggtree[2] in R version 4.3.2. Tip labels are formatted to represent "Mycobacterium_species_strain_Genbank accession".

## Supplemental References

1. Trifinopoulos J, Nguyen L-T, von Haeseler A, Minh BQ., 2016. W-IQ-TREE: a fast online phylogenetic tool for maximum likelihood analysis. *Nucleic Acids Res 44*: W232-235
2. Chen M, Luo X, Xu S, Li L, Li J, Xie Z, Wang Q, Liao Y, Liu B, Liang W, Mo K, Song Q, Chen X, Lam TT-Y, Yu G., 2025. Scalable method for exploring phylogenetic placement uncertainty with custom visualizations using treeio and ggtree. *Imeta 4*: e269
